# Epigenetic Reprogramming of the Inflammatory Response in Obesity and Type 2 Diabetes

**DOI:** 10.3390/biom12070982

**Published:** 2022-07-14

**Authors:** Federica Zatterale, Gregory Alexander Raciti, Immacolata Prevenzano, Alessia Leone, Michele Campitelli, Veronica De Rosa, Francesco Beguinot, Luca Parrillo

**Affiliations:** 1 Department of Translational Medical Science, Federico II University of Naples, 80131 Naples, Italy; federicazatterale@libero.it (F.Z.); gregoryraciti@gmail.com (G.A.R.); imma987@hotmail.it (I.P.); aleleone86@libero.it (A.L.); m.campitelli@ieos.cnr.it (M.C.); 2 URT Genomic of Diabetes, Institute of Experimental Endocrinology and Oncology, National Research Council, 80131 Naples, Italy; veronica.derosa@cnr.it

**Keywords:** epigenetics, immune system, obesity and type 2 diabetes, inflammation, adipocyte hypertrophy, WBCs, epigenetic drug therapy, EWAS

## Abstract

For the past several decades, the prevalence of obesity and type 2 diabetes (T2D) has continued to rise on a global level. The risk contributing to this pandemic implicates both genetic and environmental factors, which are functionally integrated by epigenetic mechanisms. While these conditions are accompanied by major abnormalities in fuel metabolism, evidence indicates that altered immune cell functions also play an important role in shaping of obesity and T2D phenotypes. Interestingly, these events have been shown to be determined by epigenetic mechanisms. Consistently, recent epigenome-wide association studies have demonstrated that immune cells from obese and T2D individuals feature specific epigenetic profiles when compared to those from healthy subjects. In this work, we have reviewed recent literature reporting epigenetic changes affecting the immune cell phenotype and function in obesity and T2D. We will further discuss therapeutic strategies targeting epigenetic marks for treating obesity and T2D-associated inflammation.

## 1. Introduction

The adoption of unhealthy lifestyles has led to an unprecedented prevalence of obesity and obesity-related disorders, including T2D [1]. This challenge cannot be addressed without a deeper understanding of the molecular mechanisms underlying these disorders. Along with well-investigated metabolic abnormalities, there is now accumulating evidence that the immune system also plays a major mechanistic role in the development of obesity and T2D [2].

The traditional view that immune system functions are confined to fighting pathogens has been challenged during the past decades as it became clear that the immune system plays major roles in metabolism [3]. Inflammation, for instance, is a classical immune-mediated reaction which has established as a defensive reaction against environmental insults. However, when uncontrolled, inflammation can become a chronic condition, triggering a cascade of events leading to metabolic dysfunction and contributing to a variety of metabolic disorders [4]. Indeed, chronic low-grade inflammation has been proposed as a key step in the pathogenesis of obesity-induced insulin resistance (IR) and T2D [5].

Initial evidence supporting the involvement of inflammation in obesity and T2D came from the finding that the pro-inflammatory molecule tumor necrosis factor-alpha (*TNF-α*) features increased expression in adipose tissue (AT) from obese rodents and humans with amelioration of IR after neutralization of this potent cytokine [6,7]. It is now well-recognized that excess fat mass in obese individuals is characterized by significant changes in the abundance and profile of infiltrating immune cells [8]. These changes include activation of pro-inflammatory macrophages and other immune cells that produce and secrete inflammatory cytokines and chemokines [2]. AT macrophages (ATM) from lean humans and animals are usually of the M2 type, or alternatively activated, which play a role in the attenuation and resolution of inflammation by secreting high levels of anti-inflammatory Interleukin (*IL*)*-10* [9,10]. Conversely, the AT of obese individuals harbors a larger number of M1, or classically activated, macrophages which secrete several pro-inflammatory cytokines such as *IL-6*, *IL-1β*, and *TNF-α* [11,12]. These alterations precede and contribute to the development of systemic inflammation and IR, which in turn increases the risk of T2D [2]. In line with these findings, pro-inflammatory *TNF-α* levels have been found to be higher in T2D-prone A^γ^ mice than in wild-type mice [13]. As a further example, *IL-6* levels, which are elevated in obese individuals and in patients with T2D, predict the development of these disorders [14,15]. Finally, *IL-1β*, is upregulated in islets of individuals with T2D. This master cytokine controls and is able to recruit several other cytokines, thereby implementing a broad inflammatory response in these conditions [16,17,18]. Overall, these studies indicate that components of the immune system may have specific roles in the etiology and progression of obesity and T2D. This, however, leads to the important question of what triggers the inflammatory events through the progression towards obesity and T2D.

The interactions between environmental and genetic factors appear to play a pivotal role in the onset and progression of obesity and T2D. Lifestyle factors, such as physical activity, glucose, and a high-fat diet, can modulate gene expression through epigenetic modifications [1]. Epigenetic processes control the expression of different genes, including genes involved in metabolism and the regulation of inflammatory pathways [19]. Recent studies have reported distinct epigenetic signatures in immune cells from obese individuals as well as in patients with T2D [3,20]. Thus, dysregulation of epigenetic mechanisms may represent an underlying cause of chronic inflammation in obesity and T2D. In the following section of this review, we will report on the current evidence supporting the important role of epigenetic dysregulation in determining chronic low-grade inflammation in obesity and T2D.

## 2. Epigenetics of Immune System and Its Role in the Development of Obesity- and T2D-Associated Inflammation

**Figure 1:**
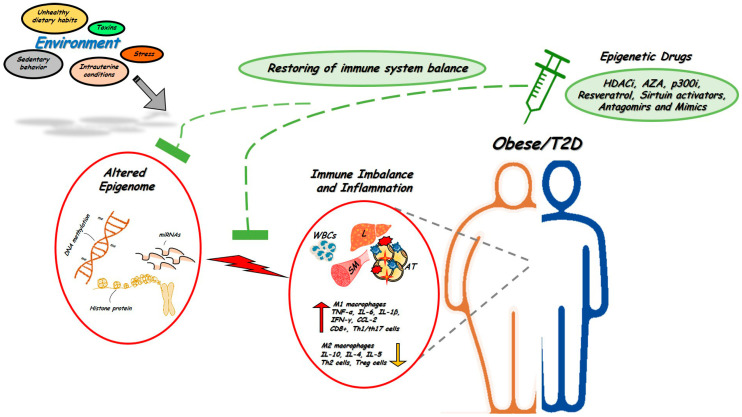
Alterations in the epigenetic signature of immune system in obesity and T2D. Adverse environmental factors could affect the epigenetic signature of immune cells to generate or aggravate tissue pro-inflammatory states and induce obesity and T2D (red circles). Targeting epigenetic mechanisms offer novel opportunities for treatment and restoring obesity- and T2D-associated inflammation (green circles). Several epigenetic drugs, including HDACi and Antagomirs or Mimics are currently being tested in vivo in human and animal models of metabolic disorders. WBCs, white blood cells; L, liver; AT, adipose tissue; SM, skeletal muscle. ↑: up-regulated expression, ↓: down-regulated expression.

The immune system is one of the most complex networks in the body. Such complexity implicates an intricate circuit of different cell types which are closely interconnected and whose functions are based upon finely-tuned epigenetic regulation. Initial events leading to epigenetic control of immune cells occur at the time of hematopoietic stem cells (HSCs) lineage commitment. HSCs give rise to common lymphoid and myeloid progenitors that are the precursors, respectively, of all lymphoid (B, T, and NK cells) and myeloid cells (monocytes, neutrophils, eosinophils, basophils and mast cells). Importantly, all of these cells differ in their epigenetic programing. Convincing evidence indeed indicates that DNA methylation, chromatin remodeling by histone tail modifications, and non-coding RNA expression, in particular miRNA, represent the most commonly epigenetic events coordinating the main features of the immune system, including cell differentiation, identity and function [21]. Moreover, since altered immune functions and onset of chronic inflammation represent early pathophysiological mechanisms accompanying the development of obesity and T2D, where the epigenome is also profoundly altered, it is possible that epigenetic abnormalities impacting on immune cells relay environmental cues in obesity and T2D (Figure 1).

### 2.1. DNA Methylation

DNA methylation consists in the addition of methyl groups to DNA. The cytosine residues occurring in cytosine–phosphate–guanine dinucleotides (CpG site) are the main targets for DNA methylation. Hypermethylation of CpG-rich regions (i.e., CpG islands) at gene promoters is commonly associated with gene silencing, while their hypomethylation often results in transcriptional activation. Methylation events are controlled by specific enzymes known as DNA methyltransferases (*DNMTs*, such as *DNMT1*, *DNMT3A*, and *DNMT3B*). DNA methylation is reversible. Also, the enzymes of the ten-eleven translocation (*TET*) methyl-cytosine dioxygenase family, namely *TET1*, *TET2* and *TET3*, catalyze the active demethylation process [1].

#### 2.1.1. DNA Methylation and Regulation of Immunity

Changes in the DNA methylation status are of major relevance to the differentiation of HSCs. Genome-wide DNA methylation profiles of differentiating blood cells have revealed that myelopoiesis and lymphopoiesis can be segregated based on cell *Methylome*, with lymphopoiesis very much depending on the acquisition of DNA methylation marks, and myelopoiesis depending much more on loss of methylation marks [22]. Consistently, the combined loss of *Dnmt3a* and *Dnmt3b* in mouse HSCs impairs their differentiation capacity, along with a profound demethylation of genes normally restricted to stem cells (HSC multipotency genes [23,24]). Most studies have shown a remarkable dynamic plasticity in methylation during human monocyte-macrophage differentiation and subsequent activation, where both the gain and loss of methylation are biologically relevant [25,26,27]. In addition, macrophage polarization is actively controlled by DNA methylation. Indeed, a complete *DNMT3b* knockdown promotes macrophage polarization to the alternatively activated M2 phenotype (anti-inflammatory), together with a decreased expression of inflammatory genes such as *TNFα*, *IL-1β*, and an altered chemotactic capacity [28].

B cell development, which is responsible for antibody-mediated immunity, also falls under the control of DNA methylation. From their early differentiation in the bone marrow to their maturation in the spleen, human B cells undergo extensive changes in their DNA *Methylome*, in up to 30% of all measured CpG sites. Early differentiation stages are accompanied by a gradual, widespread demethylation of the enhancer regions containing binding sequences of key B cell transcription factors (TFs). At variance, late differentiation shows a large-scale demethylation of heterochromatin and methylation gain at polycomb-repressed regions that do not impact on B cell specific/functional genes [29]. Furthermore, several genes involved in proliferation, migration, and nuclear factor-*κB* (*NF-κB*) signaling in B cells show an inverse correlation between promoter DNA methylation and gene expression [30]. A strict control of the DNA *Methylome* is also required for the specification of the T cell subtype as helper (CD4+) or cytotoxic (CD8+) from thymocytes. In particular, a dynamic gain and loss of methylation at the *Cd4 locus* are essential to maintain CD4 silencing in CD8+ cytotoxic cells, and to enable CD4 stable expression in T helper (CD4+) cells, respectively [31]. Concomitantly, the *Cd8b* gene, which encodes for the CD8 co-receptor, undergoes promoter hypomethylation, showing an inverse correlation with the expression in thymocytes and lymph node CD8+ T cells [32].

In addition, DNA methylation is involved in the transcriptional regulation of the C-C motif chemokine receptor type 5 (*CCR5*) gene, which is the primary chemokine receptor utilized by HIV to infect the leukocytes. In fact, monocytes and CD4+ T cells expressing high levels of *CCR5* feature an absence of methylation at the *Ccr5* promoter region [33]. Similarly, changes in the methylation status at the *T-box expressed in T cells* (*Tbet*), *Il-4*, *interferon γ* (*Ifnγ*), and *Forkhead box P3* (*Foxp3*) gene *loci* are essential for the polarization of CD4+ T cells towards the Th1, Th2, Th17, or to regulatory T cell (*Treg*) lineages [34,35,36]. The transcriptional regulation of some cytokine genes is also regulated by the active demethylation by *TET2*. Specifically, the expression of *Ifnγ*, *Il-4*, and *Il-17* is impaired in TET2^−/−^ mice [37]. *Treg*, expressing the *FOXP3* TF, play a pivotal role in the maintenance of self-tolerance within the immune system, preventing inflammatory excess and autoimmunity. Importantly, *DNMT1* actively participates with *FOXP3* to differentiate *Treg*. A loss of this enzyme in mouse T cells impairs methylation maintenance, disrupting the *Treg* transcriptional program and causing lethal immunity [38,39,40].

#### 2.1.2. DNA Methylation and the Development of Inflammation in Obesity and T2D

In obese humans, global DNA methylation levels in AT have been associated with an increased expression of specific pro-inflammatory genes, including the *MCP-1* (monocyte chemoattractant protein-1) gene [41]. The chemokine product of this gene promotes macrophage infiltration into the AT and is directly implicated in the development of IR in obesity [42]. A study by Liu et al. has reported that the methylation level at the *MCP-1* promoter decreases in peripheral blood mononuclear cells (PBMCs) from T2D patients. This epigenetic change is associated with serum *MCP-1*, blood glucose, and triglyceride levels, which may lead to a sustained increase of *MCP-1* gene expression and contribute to the development of T2D-associated inflammation [43]. Also, methylation analysis in PBMCs of T2 diabetics showed decreased levels of DNA methylation at the *IL-1β* promoter, which was accompanied by an increased gene expression. This gene may represent an epigenetic marker of chronic inflammation and T2D development, as methylation at the *IL-1β locus* significantly correlates with fasting plasma glucose and glycated hemoglobin levels in T2D subjects [44,45].

*IL-β1* mRNA production and its accumulation in the adipose tissue are also dependent on a glucose-induced activation of the *TXNIP* (*Thioredoxin Interacting Protein*) gene, which is a regulator of peripheral glucose metabolism in humans [46,47]. Interestingly, decreased skeletal muscle and blood *TXNIP* DNA methylation and increased skeletal muscle *TXNIP* expression have been reported both in subjects with increased risk of T2D and in individuals with overt disease [48]. Thus, an altered *TXNIP* methylation seems to represent a pathogenic mechanism involved in the induction of the inflammatory response accompanying T2D.

In another study, obese women were reported to exhibit higher plasma *TNF-α* levels accompanied by lower methylation at its promoter, linking an altered DNA methylation to systemic inflammation in obesity [49]. Similarly, Korean women with an excessive body weight were shown to feature aberrant methylation at the *IL-6* gene promoter in blood cells [50]. Likewise, the MENA (Methyl Epigenome Network Association) project has reported a strong association between altered DNA methylation in PBMCs and waist circumference in specific methylation sites of several inflammatory genes, including *GRIK3* (*Glutamate ionotropic Receptor Kainate type subunit 3*), *ZC3H12D* (*Zinc Finger CCCH-Type Containing 12D*) and *TREM-1 (Triggering Receptor Expressed on Myeloid Cells 1*) [51]. At variance, Simar et al. have reported no correlation between global DNA methylation in PBMCs (consisting of both lymphocytes and monocytes) and obesity or T2D [52]. However, they found that both obese and T2 diabetic individuals are characterized by an increased global DNA methylation in B and NK cells when compared to lean subjects, and this altered methylation profile is gene- and cytosine-specific.

The *DNMTs* also contribute to altered immune-cell function in metabolic diseases. For example, *DNMT3B* expression promotes M1 macrophage polarization and AT inflammation by increasing DNA methylation at the *PPARγ1* promoter, leading to *PPARγ1* downregulation. *PPARγ1* has anti-inflammatory properties and favors the transcription of M2 macrophage-associated genes. In addition, *DNMT3B* is upregulated in in vitro isolated ATM treated with saturated fatty acids, whose plasma levels are increased in obesity. Deletion of this enzyme in macrophages not only enhances M2 macrophage polarization but also restores insulin sensitivity in adipocytes [28,53]. Myeloid *DNMT1* inhibition leads to a decreased macrophage *PPARγ1* promoter DNA methylation, which, in turn, promotes an increased ATM alternative activation and decreased inflammation in obese animals [54]. *DNMT1* is also responsible for the hypermethylation of the adiponectin promoter in obese mice and humans, and causes decreased expression of this anti-inflammatory cytokine, worsening of IR and obesity-associated inflammation [55].

Taken together, these findings indicate that obesity and T2D are intimately linked to a remodeling of the DNA *Methylome* both in WBCs and in specific immune cell sub-populations, contributing to the altered immune function reported in these metabolic disorders. In the future, changes in the *Methylome* of immune cells might be used for the prediction of individual disease risk and as targets for treatment.

### 2.2. Histone Modifications

In the eukaryotes, the basic unit of chromatin is the nucleosome, which is a dynamic structure of 147 base pairs of DNA wrapped around an octamer of core histone proteins (two copies of each histone H2A, H2B, H3, and H4). Each histone has an *N*-terminal tail, that protrudes from the nucleosome, which can be decorated with various post-translational modifications (PTMs), including acetylation, methylation, phosphorylation and ubiquitination. These PTMs, which are positioned or removed by specialized modifying enzymes, either loosen (enabling access for transcriptional machinery) or lock (preventing access) the packing of the DNA around the histones, thus regulating gene expression [56].

#### 2.2.1. Histone Modifications in Immunity

Parallel to changes in the DNA methylation pattern, histone modifications also represent key regulators of genome structure and gene expression which affect the immune response [57]. Upon activation, naïve CD4+ T cells can differentiate into a variety of T cell subsets, including Th1 or Th2 lineages. CD4+ T cell lineage commitment correlates with dynamic changes in histone PTM deposition at the gene *loci* associated with directing subset specific CD4+ T cell effector function [58]. For instance, this is the case for the expression of *Ifnγ* in the CD4+ T cell. The *Ifnγ locus* shows a strong enrichment of the repressive mark H3K27me3 (tri-methylation of histone H3 at lysine 27) upon Th2 differentiation and in naïve CD4+ T cells, while the same *locus* features increased levels of the permissive histone mark H3K4me2 during differentiation of Th1 cells [59]. A similar scenario also occurs in directing the naïve CD8+ T cell fate. In particular, the deacetylated histone H3, which is a marker of open chromatin, increases as naive CD8+ T cells develop into memory T cells. Interestingly, this modification has been proposed as a marker for assessing memory T cell functionality, since memory T cells with defective rapid recall ability have less deacetylated histone H3 than their fully functional counterparts [57].

The development and activation of different B-cell subsets are also associated with the acquisition of a distinct pattern of histone marks [60]. For example, analysis of histone lysine methylation in resting cells and activated cells has shown that methylation of histone H3 at lysines 4 (H3K4), 9 (H3K9), 27 (H3K27) and histone H4 at lysine 20 is markedly reduced in quiescent B lymphocytes as compared with activated B cells [61]. The potential role of histone changes in controlling B cell function has been further documented by the ability of histone deacetylase inhibitors (HDACi) to affect B cell proliferation, survival and differentiation in a lupus-prone mouse model [62].

Histone change events are also associated with the determination of macrophage fate. For instance, the enzyme SMYD-3 (SET and MYN domain), an H3K4 methyltransferase, has been shown to positively regulate M2 polarization. Its activity was increased in human macrophages upon exposure to M-CSF (macrophage colony stimulating factor) in combination with *IL-4* and *IL-13*, while it was decreased upon exposure to M1 stimulation [63]. Another typical example is represented by *JMJD3* (*Jumonji* Domain Containing Protein D3), a H3K27 demethylase which has been recognized to control M2 macrophage markers. *JMJD3* promotes di- and tri-demethylation of H3K27, leading to an activation of *Arg1* (arginase) and *Irf-4* (interferon regulatory factor 4), among other M2 markers [64]. Histone acetylation can further influence macrophage polarization. HDAC3, for example, promotes M1 macrophage polarization by acting as an epigenomic brake in macrophage alternative activation [65], while HDAC2, recruited by *Tet2*, represses transcription of *IL-6* via histone deacetylation, promoting a resolution of the inflammation [66]. Moreover, HDAC9 deletion results in downregulation of inflammation-related genes and in polarization of macrophages towards the M2-like phenotype through a strong enrichment of acetylated H3 at *ABCA1* (*ATP binding cassette subfamily A member 1*), *ABCG1* (*ATP binding cassette subfamily G member 1*), and *PPARγ* (*Peroxisome proliferator-activated receptor gamma*) macrophage promoters [67]. Macrophage trafficking is also under the control of histone marks. Indeed, H3K9ac and H3K4me3 regulate the expression of the *CCL2* and *CCL3* chemokines (*C-C motif chemokine ligand*) and their receptors [68].

Thus, the activation of immune cells is dependent on a complex network of transcriptional regulators whose *loci* are characterized by histone marks which dynamically switch from a repressive to a permissive state in order to enable a prompt response to external stimuli. Histone modifying agents (e.g., HDACi) are currently under extensive investigation for their potential efficacy in inflammatory disorders.

#### 2.2.2. Histone Marks in Obesity and T2D

A number of changes in histone PTMs with impact on inflammatory pathways have been reported in obesity and T2D [69]. Culturing monocytes in the presence of high glucose levels leads to increased H3 hyperacetylation at the promoters of *NF-κB*-dependent inflammatory genes, mimicking findings in T2D patients. Consistently, increased levels of histone acetylation at the promoter of *TNF-α* and *COX-2* (*Cyclooxygenase-2*) have been observed upon treatment of monocytes with HDACi [70]. The histone methyltransferase SET7/9 (*SET domain containing 7*) is an essential co-activator of *NF-κB* and mediates methylation at H3K4. In human monocytes, SET7/9 enhances the recruitment of *p65*, which acts as a trigger for the induction of expression of *NF-κB*-related genes. In monocytes exposed to TNF-α, SET7/9 deletion not only inhibits inflammatory genes, including *TNF-α*, *MCP-1* and *IL-6*, but also attenuates diabetes-induced inflammatory processes [71]. Importantly, Panemi et al. have shown that in PBMCs from T2 diabetics, SET7/9 increases methylation of H3K4 at the *NF-κB p65* promoter leading to an higher expression of *MCP-1*, *ICAM-1* (*Intercellular Adhesion Molecule 1*) and *COX-2* [72]. Gallagher et al. also reported that, at the *IL-2* gene promoter, H3K27me3, a repressive histone methylation mark, is decreased in bone marrow progenitors, which is passed to wounded macrophages in a murine model of glucose intolerance. These epigenetically pre-programming generates poised macrophages in peripheral tissues and negatively impact on wound repair. Decreased methylation at H3K27 is accomplished by *Jmjd3*. Its inhibition enhances H3K27me and *IL-12* levels, providing an innovative, though potential, epigenetic approach for wound healing in T2D [73]. Furthermore, in human monocytes, hyperglycemia induces decreases in the histone repressor mark H3K9me3 at *IL-6* promoter, accompanied by increased *IL-6* expression and exacerbation of inflammation.

Obese individuals exhibit reduced levels of HDAC4 in PBMC and AT, which is negatively correlated with circulating levels of the pro-inflammatory chemokine *RANTES/CCL5*. Intriguingly, in the same subjects HDAC4 expression was restored after physical exercise, which makes HDAC4 an appealing, though potential, target for mitigating obesity-associated inflammation [74]. In addition, gene expression analyses have revealed a correlation between different HDACs (HDAC2, 4, 5, and 6), adiposity parameters, and obesity-associated inflammatory events in subcutaneous and visceral fat depots of obese women [75]. Of note, dietary-induced and genetic mouse models of obesity display a decreased level of KDM1A (Lysine Demethylase 1A), a histone demethylase, in AT, which correlates with an increased expression of pro-inflammatory genes [76].

It has also been reported that treatment of hepatocytes with free fatty acid upregulates the expression of the Brahma-related gene1 (Brg1), which, in turn, promotes inflammation by increasing permissive histone acetylation at the promoter regions of the IL-1, IL-6 and MCP-1 genes. Depletion of Brg1 attenuates the release of pro-inflammatory mediators in the liver and significantly ameliorates obesity-related hepatic inflammation [77]. In vitro lipopolysaccharide (LPS)-treated hepatocytes display an increased histone acetylation of H3K9/K18Ac at the inflammatory TNF-α and Ccl2 gene loci [78]. In addition, the H3 deacetylase SIRT (Sirtuin)-1 exhibits a decreased expression in the AT of obese patients, resulting in enhanced macrophage recruitment through chemoattractant and cytokine secretion [79]. On the same line, obese children with IR show downregulation of both SIRT1 and 2 in PBMCs [80,81], while administration of hypocaloric diets in obese individuals results in SIRT1 and 2 upregulation [82]. The crucial role of histone modifying enzymes (e.g., HDACs) and their inhibitors (e.g., HDACi) in the regulation of inflammatory events makes them ideally suited targets for the treatment of inflammatory diseases, such as obesity and T2D.

### 2.3. microRNAs

MiRNAs are single-stranded RNAs containing 20–24 bases. They primarily act as post-transcriptional repressors by targeting mRNA 3′-untranslated regions and stimulating their degradation and translation repression [83,84]. In mammals, miRNAs are predicted to directly control up to 60% of all protein-coding genes. In addition, a specific miRNA is able to regulate more than one target mRNA even within the same signaling pathway, thereby creating different steps of gene regulation [85].

#### 2.3.1. Role of microRNAs in the Immune Response

Emerging evidence indicates that miRNAs play very important roles in immune cells, including HSC differentiation and maturation [86]. For example, the combined deletion of *miR-23a* and *miR-23b* leads to an improper hematopoietic progenitor cell production and differentiation in mice [87]. Other miRNAs which are responsible for regulating hematopoietic lineage differentiation include *miR-181*, *miR-223*, and *miR-142* [88]. Furthermore, Shangqin Guo et al. [89] have reported an essential role of Dicer (the enzyme responsible for miRNA biogenesis [90]) in HSC maintenance and have identified *miR-125a* as a positive regulator of the HSC regeneration of hematopoiesis, at least in part, by reducing the pro-apoptotic protein Bak1 (Bcl2 antagonist/killer 1 [89]).

MiRNAs finely tune the differentiation and activation of B cells. The expression of *miR-1246* in normal B cells regulates cell responsiveness by upregulating the expression of several molecules that allow B cells to interact with T cells and provide an effective immune response [91]. *MiR-16* and *miRNA-let-7c* modulate the ability of CD4 T cells to discriminate between activating and energizing stimuli by targeting the mTOR (mechanistic target of rapamycin kinase) components *Mtor* and *Rictor* (*RPTOR independent companion of mTOR complex 2* [92]). Also, Dicer^−/−^ CD4+ T cells favor Th1 cell differentiation via an increased expression of the Th1-specific transcription factor *Tbx21* (*T-box transcription factor 21*) and production of *IFNγ* [93]. At variance, *miR-29a* and *miR-29b* negatively regulate Th1 differentiation by repressing *Tbet* and *Eomes* (*Eomesodermin*), two transcription factors known to induce Th1 lineage and *IFNγ* production [93]. Several studies have further identified differentially expressed miRNAs that provide M1 and M2-polarized macrophages signatures [94]. In particular, M1-polarized macrophages are feature a high expression of *miR-155*, *miR-9*, *miR-146a,* and *miR-19*, whereas higher levels *of miR-26a-2-3p* and *let-7c* identify M2-polarized macrophages [95]. *MiR-127* [96] and *miR-125b* [97] also induce M1 polarization by targeting *Bcl6* (*B cell leukemia/lymphoma 6*) and *IRF4* (*Interferon regulatory factor 4*), respectively, with a consequent increase in the expression of pro-inflammatory cytokines.

The NF-κB family of transcription factors coordinates the expression of an array of genes involved in the inflammatory responses (e.g., *TNF-α*, *IL-1β*, and *IL-6*). Interestingly, a specific subset of miRNAs, including *miR-146a*, *miR-155*, and *miR-9*, are directly controlled by this inflammatory signalling pathway, and in several cases these miRNAs act as a feedback control system of the *NF-κB*-dependent immune response by regulating its key members [94]. Thus, the intricate miRNA network represents a pivotal player which actively participates in the modulation of immune cell function and inflammatory response.

#### 2.3.2. MicroRNAs and the Development of Inflammation in Metabolic Disorders

A growing body of evidence supports the hypothesis that specific miRNAs regulate obesity- and T2D-associated inflammation. *Mir-320*, involved in the modulation of insulin signaling, is increased in the AT from obese individuals and exhibits a positive correlation with elevated levels of several inflammatory markers, including *TNF-α*, *NF-κB*, and *IL-6*. Interestingly, adipocyte knockdown of *mir-320* induces downregulation of these inflammatory factors, suggesting that selectively inhibiting *mir-320* represents a strategy to ameliorate inflammation response in obesity settings [98]. Likewise, in obese patients, a reduced expression of *mir-126* and *mir-193b* is associated with upregulation of the chemokine *CCL2*, while their overexpression attenuates *CCL2* production in human monocytes, thus affecting immune cell recruitment and AT inflammation [99]. By using an integrated computational strategy, Zhang et al. have identified 23 active miRNA-TF gene regulatory pathways which are significantly associated with obesity-related inflammation. Among these, *mir-193b* is of particular interest since its expression strongly correlates with signaling through the *TNF-α* pathway [100]. Several exosomal miRNAs are also differentially expressed in subcutaneous and visceral AT from patients with obesity. Notably, these *miRNAs* preferentially target genes involved in key inflammatory processes. It is proposed that these *miRNAs* and their target genes represent novel therapeutic targets for metabolic disease management [101]. Besides, the expression of adipocyte-secreted *mir-34a* has been reported to increase progressively with the development of dietary obesity, whereas its adipose-selective ablation leads to macrophage polarization into the anti-inflammatory M2 phenotype, and protects mice from inflammation and IR caused by dietary stress [102].

It has also been reported that several anti-inflammatory miRNAs, such as *mir-1934*, *mir-532-5p* and *mir-146a* are downregulated in overweight and obesity, which may mechanistically contribute to the chronic inflammatory state associated with these disorders [103,104,105,106]. T2D patients are associated with decreased PBMC expression of *mir-146a*, which is correlated with IR, poor glycemic control, and expression of several pro-inflammatory genes with increased plasma levels of *TNF-α* and *IL-6* [107]. Analysis of plasma miRNA expression profiles in patients with T2D has revealed decreased levels of a set of miRNAs involved in inflammatory pathways [108]. For example, *mir-15a* shows a downregulation under hyperglycemic conditions, while its overexpression attenuates the pro-inflammatory signaling of *IL-1β*, *TNF-α* and *NF-κB* [109]. Anti-inflammatory function role has also been proposed for *mir-20b* and *mir-29b*, as these miRNAs target proteins responsible for the differentiation of the Th17 and Th1 T-helper cells, which produce pro-inflammatory cytokines [110,111]. Interestingly, deregulation of *mir-29b* along with that of *mir-15a* precedes T2D onset [108].

*Mir-103* and *mir-143* are implicated in adipogenesis dysfunction and IR, and feature increased expression in PBMCs from murine models of pre-diabetes and T2D [112,113]. Increased expression of several members of the *mir-200* family is associated with T2D-related vascular inflammation. In particular, *mir-200b*, *mir-200c*, and *mir-492* induced overexpression of *COX-2* and *MCP-1* in vascular smooth muscle cells (VSMCs) from diabetic mice by repressing the *Zeb1* (*Zinc Finger E-Box Binding Homeobox 1*), which negatively regulates inflammatory genes [114]. Additionally, *mir-504* was also found to be increased in VSMCs from diabetic mice and shown to reduce the expression of two target genes, namely *Grb10* (*Growth factor receptor bound protein 10*) and *Egr2* (*Early growth response 2*), whose downregulation facilities the expression of a set of inflammatory genes in mice [115].

These studies underline the involvement of specific miRNAs in the development of obesity- and T2D-related inflammation. Some of these miRNAs may not only become promising tools for treatment of obesity and T2D, but may also be used as biomarkers for the early detection and staging of metabolic disorders. The use of miRNAs as diagnostic and therapeutic tools in clinical settings holds several advantages, including their stability and easy detectability in body fluids. Nonetheless, factors such as a lack of standardized measurements and/or an absence of a disease-specific miRNA database make this attractive goal still challenging.

### 2.4. Adipocyte Hypertrophy: A Paradigm of Epigenetic Derangement in Chronic Inflammation

Obesity and a family history of T2D are major predisposing factors for the development of T2D [116]. In both of these conditions an inappropriate expansion of subcutaneous adipose cells, has been reported [117]. Key characteristics of this abnormality, in addition to enlarged adipocytes, is the impaired adipocyte differentiation, presence of senescent fat cells, fibrosis and, most importantly, evidence of inflammatory state [118]. Notably, hypertrophic obesity has long been known to be an independent predictor of T2D [119,120]. Research by our group has recently identified epigenetic processes as key mechanisms in the development of these abnormalities. In these studies, we have also shown that adipocyte hypertrophy in first-degree relatives of T2 diabetics (FDR) and in obese subjects is accompanied by epigenetic alterations that affect genes responsible for the development of a pro-inflammatory state (Table 1 and Table 2). This makes of adipocyte hypertrophy a paradigm of epigenetic-associated inflammatory disease.

We have recently reported that the *Methylome* profile of subcutaneous adipocyte precursor cells (APCs) from individuals who are FDR is associated with extensive hypomethylation events affecting major molecular pathways involved in the transduction signals controlling inflammation processes [117]. The strongest hypomethylation signal was identified at the *PTPRD* (*Protein Tyrosine Phosphatase Receptor Type D*) gene, which marks hypertrophic obesity in FDR. This methylation pattern was also replicated in obese individuals. *PTPRD* belongs to the protein tyrosine phosphatase (*PTP*) superfamily and has been identified as a T2D susceptibility gene [126,127]. Importantly, *PTPs* have been associated with multiple immune-related disorders in both animal models and in human diseases [128]. Even more recently, Spinelli et al. have reported that in healthy FDR subjects, the senescence-related *ZMAT3* (*Zing Finger Matrin-Type 3*) gene exhibited a decreased methylation, which was responsible for its upregulation [121]. *ZMAT3* regulates *p53* which in turn acts as a molecular link between pathways involved in inflammation and IR in the AT [13,129]. Of note, APCs from these FDR subjects displayed an enhanced secretion of several pro-inflammatory factors, such as *IL-6*, *MCP-1*, *RANTES*, and *IL-8*, which further appeared to be connected with *ZMAT3* hypo-methylation [121].

**Table 2:** Epigenetic alterations affecting inflammatory genes/pathways in mouse hypertrophic obesity models.

Study Model	Epigenetic Marks	Position	Processes	Medical Condition	Species	Ref
AT from Diet-induced Obesity model	DNA Hyper-methylation	*Hoxa5*	Hox Gene Family, Adipogenesis, AT Macrophage Genes	Obesity, Impaired Glucose Metabolism, AT Inflammation	Mouse	[130]
AT from Diet-induced Obesity model	-	*Hoxa5*	ER Stress Signalling pathways, M2 Macrophage Polarization	Obesity, Impaired Glucose Metabolism, AT Inflammation	Mouse	[131]
PMCs from Diet-induced Obesity Model	-	*Zfp423*	Nf-κb Inflammatory pathway, ATM accumulation, LPS-induced Inflammation	Obesity, AT Inflammation	Mouse	[132]
AT from Diet-induced Obesity and from Obese subjects	DNA Hyper-methylation	*Ankrd26*	Adipocyte pro-inflammatory secretion, *Il-8*, *Mcp-1*, Rantes/Pro-inflammatory profile in AT	Obesity, Impaired Glucose Metabolism, Adiposity, AT Inflammation	Mouse	[133]

AT, adipose tissue; APC, adipocyte precursor cells; PBL, peripheral blood leukocytes; FDR, first-degree relatives of type 2 diabetics; ER, endoplasmic reticulum; PMCs, perivascular mesen-chymal cells; ATM, adipose tissue macrophages; LPS, lipopolysaccharides.

Not only loss of DNA methylation, but also the increase in methylation levels at specific genes and genomic regions is biologically relevant in shaping the phenotype of individual conditions. For instance, we have found that, in FDR and obese individuals, hypertrophic obesity is associated with increased methylation levels at the promoter of the *HOXA5* (*Homeobox A5*) gene. In human pre-adipocytes, *HOXA5* silencing leads to an impaired adipogenesis along with inappropriate activation of *WNT*-signaling (*Wingless-related integration site*) genes, which leads to local and systemic inflammation in AT [122]. Importantly, *Hoxa5* reduced inflammatory cytokine secretion and promoted an increased number of M2 macrophages in the AT of high-fat diet mice [131], whereas its methylation-dependent silencing was associated with an elevated expression of macrophage marker genes, including *F4/80*, *Cd68* (*Cluster of differentiation 68*) and *Mcp-1*, and a disturbed glucose metabolism [130]. Similarly, we have observed massive hypermethylation at the promoter of the human *ZNF423* (*Zinc Finger Protein 423*) gene, which accounted for the reduced *ZNF423* expression observed in hypertrophic obesity [123]. *Zfp423* has been identified as a transcriptional break on *NF-κB* signaling. In perivascular mesenchymal cells Z*fp423* suppresses inflammatory signaling and attenuates metabolic inflammation in the AT of diet-induced obesity, while its ablation, facilitating the activation of *NF-κB* signaling, exacerbates AT macrophage accrual and promotes AT dysfunction [132]. Furthermore, we demonstrated that diet-induced obesity in mice led to a hyper-methylation of the *Ankrd26* (*Ankyrin repeat domain containing 26*) gene promoter, which further contributed to increased secretion of pro-inflammatory factors in AT [133]. This gene has been previously associated with the development of obesity and T2D. Consistently, in the PBL from obese individuals, epigenetic silencing of the *ANKRD26* gene by promoter hyper-methylation correlated with increased levels of several inflammatory factors including *IL-6*, *IL-8*, and *RANTES* [124].

In addition to DNA methylation changes, alterations in miRNA expression and histone modifications also contribute to the inflammatory state accompanying hypertrophic AT changes. Analysis of *miRNome* profile in APC from FDR identified a set of differentially expressed miRNAs featuring a strong enrichment in pro-inflammatory pathways, which may be relevant to the hypertrophic changes of FDR individuals [125]. Several of these miRNAs targeted the *Insulin-like Growth Factor 2* (*IGF2*) gene, whose dysregulation induces inflammatory disease [134]. Finally, in FDR subjects, altered histone marks were identified at genes related to mitochondrial dysfunction (unpublished data), a major cause of adipose tissue inflammation [135].

All together, these findings strengthen the role of epigenetic mechanisms in inflammation-related metabolic disorders, and reveal the existence of different layers of epigenetic dysregulation which determine subcutaneous adipocyte hypertrophy.

## 3. Epigenetic Changes as Anti-Inflammatory Targets for Treatment of Metabolic Disorders

In the recent past, significant progress has been made in the understanding and treatment of metabolic disorders. However, it appears that traditional medications are not able to prevent the progression of these disorders, indicating that additional therapeutic strategies are needed. In addition, epigenetic changes are often reversible making them a promising therapeutic target [1]. Since the evidence presented in the previous section of this review indicates that aberrant epigenetic variations lead to dysregulation of the inflammatory pathways involved in obesity and T2D, immunomodulatory therapies targeting the epigenetic profiles of obesity and diabetes may offer additional benefits for the treatment of these disorders. Indeed, patients with metabolic diseases receive beneficial effects on the glucose metabolism and IR upon treatment with anti-inflammatory drugs [136,137,138].

Several epigenetic modifiers have been identified, including HDACi, DNMT inhibitors (e.g., *azacytidine*) and novel miRNA-based drugs (Figure 1, [139]). HDACi have been successfully used in the treatment of several inflammatory disorders such as cancer, immune and infectious diseases [140]. These drugs have been proposed to display beneficial effects on T2D and obesity as well. For instance, the HDACi sodium phenylbutyrate ameliorates IR and the β-cell dysfunction induced by prolonged elevation of free fatty acid levels in obese individuals, by reducing inflammatory events associated to endoplasmic reticulum stress [141]. Lewis et al. have reported that oral administration of HDACi ITF2357 increases β-cell survival and enhances insulin secretion, while reducing the production and/or activities of pro-inflammatory chemokines [142]. Similarly, HDAC3 inhibition improves inflammation, hyperglycemia and insulin secretion in obese diabetic rats [143]. Resveratrol, which features HDACi activity, inhibits pro-inflammatory gene expression, significantly ameliorating glucose control and insulin sensitivity in T2D patients [144]. Resveratrol is also a potent activator of SIRT1, and its supplementation decreases systemic inflammation markers, such as *IL-6* and *TNF-α*, along with increasing insulin sensitivity in obese humans, with no adverse effects [145]. The histone acetyltransferase (HAT) p300 plays an important role in the activation of *NF-kB* target genes [146]. Thus, p300 targeting may specifically prevent inflammatory events. Curcumin, for instance, has been identified as a specific inhibitor of p300, with several studies showing anti-inflammatory properties and supporting its use in treatment of both IR and T2D [147]. Recently, two other competitive inhibitors of p300, C646, and A-485, have been identified [148,149]. These molecules have been effectively used in cancer-related inflammation [150,151], suggesting that these compounds may also have great therapeutic potential in preventing an altered immune response in metabolic conditions.

The DNA methyltransferases inhibitor *5-azacytidine* (AZA) and its derivate *5-aza 2′ deoxycytidine* (DAC) have also been proposed as potential candidates for treatment of inflammatory disorders, including obesity and T2D. Both of these drugs prevent pro-inflammatory events by fostering generation of anti-inflammatory *Treg* cells [152]. Therefore, promoting *Treg* production by targeting DNA methylation could offer a promising novel treatment option.

However, concerns related to the use of epigenetic drugs in metabolic dysfunction need to be addressed. The low specificity and global action of these agents could potentially compromise host immunity, as described in [153,154]. Moreover, the number of epigenetic changes that must be reversed is uncertain and, given that a single molecule regulates several signaling pathways and has different biological targets, one may also anticipate multiple side effects [155]. Another issue that needs to be addressed is the dynamic nature of epigenetic changes. While making epigenetic targets pharmacologically attractive, their plasticity may also render them too unstable.

Recently, miRNAs have gained increasing interest as research tools and as a potential treatment options of several diseases [156]. Novel miRNA-based therapies have been proposed to represent valuable experimental strategies for the treatment of cancer and infectious diseases [157,158]. They are commonly based on the adoption of antagomirs, which efficiently and specifically silence endogenous miRNAs or RNA small molecules which mimic endogenous miRNA function (i.e., miRNA mimics). Thus, while several issues still need to be addressed (e.g., target specificity and delivery, reduction of off-target effects or long-term safety concerns) miRNA-based therapy has, in the near future, the potential for representing a very appealing strategy for rescuing immune system function in obesity and T2D.

## 4. Conclusions

The studies reviewed in the present work indicate that aberrant changes in the epigenome represent one of the central underlying mechanisms of the immune function perturbation involved in the onset and progression of common metabolic disorders. The dynamic and reversible nature of epigenetic marks creates both challenges and unique opportunities for the development of strategies for the treatment and prevention of the inflammatory derangement associated with obesity and T2D. Indeed, several classes of epigenetic drugs, including HDAC and p300 inhibitors, SIRT1 activators and anti-miRNA molecules, have already demonstrated promising results in the treatment and management of these disorders. Despite this encouraging scenario, significant investments are required before epigenetic therapies can be adopted in clinical practice.

## Figures and Tables

**Table 1:** Epigenetic alterations affecting inflammatory genes/pathways in human hypertrophic obesity models.

Study Model	Epigenetic Marks	Position	Processes	Medical Condition	Species	Ref
SAT APC and PBL from FDR, PBL from Obese subjects	DNA Hypo-methylation	Global/*PTPRD*	Inflammation by chemokine and cytokine pathway and Adipogenesis/PTPs	SAT Hypertrophy, Familiarity for T2D, Obesity	Human	[117]
SAT APC from FDR	DNA Hypo-methylation	*ZMAT3*	Pro-Inflammatory markers: IL-6, MCP-1, RANTES, IL-8, MIP1b/Senescence and Aging	SAT Hypertrophy, Familiarity for T2D	Human	[121]
SAT APC and PBL from FDR, PBL from Obese subjects	DNA Hyper-methylation	*HOXA5*	WNT-Signaling Pathway, Adipogenesis	SAT Hypertrophy, Familiarity for T2D, Obesity	Human	[122]
SAT APC from Human Hypertrophic Obesity	DNA Hyper-methylation	*ZNF423*	Adipogenesis	SAT Hypertrophy	Human	[123]
PBL from Obese Subjects	DNA Hyper-methylation	*ANKRD26*	Adipocyte Pro-Inflammatory Markers: IL-1β, IL-6, IL-12, IL-8, IP-10, MIP-1α, MIP-1β, RANTES	Obesity, Cardio- metabolic Risk	Human	[124]
SAT APC and PBL from FDR	Deregulation of miRNA expression	*Mir-23a-5p*, *mir193a-5p*, *mir-193b-5p*	Pro-inflammatory pathway, adipogenesis, IGF2 signaling	SAT Hypertrophy, Familiarity for T2D	Human	[125]

SAT, subcutaneous adipose tissue; APC, adipocyte precursor cells; PBL, peripheral blood leukocytes; FDR, first-degree relatives of type 2 diabetics; ER, endoplasmic reticulum; PMCs, perivascular mesenchymal cells.
